# Effect of overexpressing *nhaA* and *nhaR* on sodium tolerance and lactate production in *Escherichia coli*

**DOI:** 10.1186/1754-1611-7-3

**Published:** 2013-01-29

**Authors:** Xianghao Wu, Ronni Altman, Mark A Eiteman, Elliot Altman

**Affiliations:** 1 BioChemical Engineering Program, College of Engineering, University of Georgia, Athens, GA, 30602, USA; 2 Department of Biology, Middle Tennessee State University, Murfreesboro, TN, 37132, USA

**Keywords:** Sodium tolerance, *nhaA*, *nhaR*, Lactate, Lactic acid, Fed-batch fermentation

## Abstract

**Background:**

Like other bacteria, *Escherichia coli* must carefully regulate the intracellular concentration of sodium ion (Na^+^). During the bacterial production of any organic acid, cations like Na^+^ invariably accumulate during a process which must maintain a near neutral pH. In this study, the *E. coli nhaA* gene encoding the Na^+^/H^+^ antiporter membrane protein and the *nhaR* gene encoding the NhaA regulatory protein were overexpressed in wild-type *E. coli* MG1655 and in MG1655 *pflB* (ALS1317) which lacks pyruvate formate lyase activity and thus accumulates lactate under anaerobic conditions.

**Results:**

Expression of either the *nhaA* or *nhaR* gene on the high copy inducible expression vector pTrc99A caused a significant reduction in the growth rate of MG1655. No change in growth rate was observed for MG1655 or ALS1317 for Na^+^ concentrations of 0.75–0.90 M when the medium copy pBR322 plasmid was used to overexpress the two genes. In a fed-batch process to produce the model acid lactate with NaOH addition for pH control, lactate accumulation ceased in MG1655, MG1655/pBR322, MG1655/pBR322-*nhaR* and MG1655/pBR322-*nhaA* when the concentration reached 55–58 g/L. In an identical process lactate accumulation in MG1655/pBR322-*nhaAR* did not terminate until the concentration reached over 70 g/L.

**Conclusions:**

Although overexpression the genes did not improve growth rate at high Na^+^ concentrations, the overexpression of *nhaA* and *nhaR* together led to a 25% increase in lactate production. Thus, the observed (absence of) impact that these genetic modifications had on growth rate is a poor indicator of their effect on acid accumulation. The overexpression of *nhaAR* did not cause faster lactate production, but permitted the culture to continue accumulating lactate at 10% greater Na^+^ concentration.

## Background

The sodium ion (Na^+^) plays an important role in cellular function and bioenergetics, and cells rely on a very efficient homeostatic mechanism to maintain the intracellular sodium concentration [1]. *E. coli* typically maintains a sodium gradient across the cell membrane (Na^+^_in_ < Na^+^_out_) via two Na^+^/H^+^ antiporter membrane proteins, NhaA and NhaB [2,3]. These antiporters exchange Na^+^ or Li^+^ for H^+^[4,5] and are driven by the electrochemical proton gradient (i.e., H^+^_out_ > H^+^_in_) generated by the primary proton pumps. NhaA encoded by *nhaA*[6] is absolutely required for survival in the presence of 700 mM Na^+^ at a pH of 6.8 [2]. NhaB alone confers limited sodium tolerance to cells, though this protein becomes essential when the lack of NhaA expression limits growth [7].

During exponential growth, *nhaA* responds to Na^+^ by increasing transcription via a Na^+^-specific regulatory system mediated by the positive regulator protein NhaR encoded by *nhaR*[8]. The expression of *nhaR*, which is located just downstream of *nhaA*, is induced specifically by Na^+^. The NhaR protein regulates *nhaA* expression by undergoing a conformational change upon Na^+^ binding, which modifies the NhaR-*nhaA* contact points [8]. Hence, NhaR appears to be both a sensor and a transducer of the Na^+^ signal.

The accumulation of cations like Na^+^ is germane for bioprocesses which generate an organic acid. If an optimal pH is to be maintained for the continued formation of such products, a base such as NaOH must be added into the system leading to the accumulation of cations. Previous results which show that addition of the osmoprotectant betaine improves organic acid accumulation [9] suggest that organic acid accumulation in the model organism *E. coli* may be limited by the accumulation of cation rather than by the acid anion itself. Acid accumulation is readily achieved by *E. coli* through a *pflB* knockout which lacks pyruvate formate lyase activity and although unable to grow, generates lactate under anaerobic conditions [10]. The objective of this study was to examine the effect of overexpressing *nhaA* and *nhaR* on growth and lactate production in *E. coli*.

## Results

### Overexpression of nhaA or nhaR using the high-copy inducible expression vector pTrc99A

The NhaA protein serves as a Na^+^ extruding antiporter, while NhaR serves as a positive regulator for the expression of the *nhaA* gene. We first wanted to test whether increased expression of *nhaA* or *nhaR* would increase the maximum specific growth rate of *E. coli* in the presence of high Na^+^ concentration. Although previous studies have considered the effect of *nhaA* overexpression (only) on growth [11,12], these studies used complex media, focused on changes in the promoter to affect expression level, and did not explicitly measure growth rate at both low and high Na^+^ concentration. We examined the effects of overexpressing *nhaA* and *nhaR* in the widely used pTrc99A high-copy number inducible expression vector, which contains the origin of replication from pUC8 [13].

**Figure 1:**
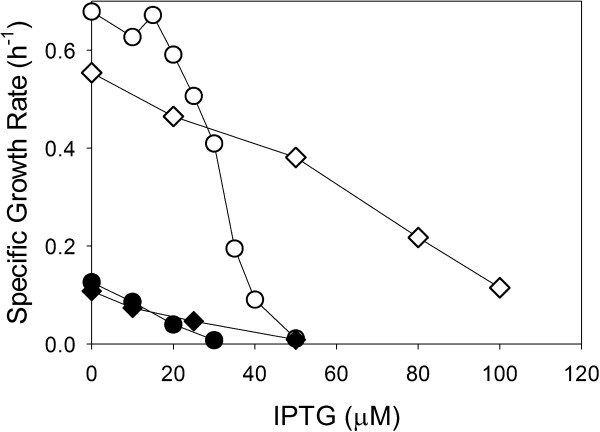
**Maximum specific growth rate (μ**_**MAX**_**) of MG1655/pTrc99A-*nhaA *(○, ●) and MG1655/pTrc99A-*nhaR *(◊, ♦) at low (<0.10 M) Na**^**+ **^**concentration (hollow symbols) and at 0.75 M Na**^**+ **^**concentration (solid symbols).** The specific growth rate of MG1655 was about 0.69 h^-1^ with <0.10 M Na^+^ and 0.17 h^-1^ at 0.75 M Na^+^ concentration.

First, we determined how IPTG induction affected the growth rates of MG1655/pTrc99A-*nhaA* and MG1655/pTrc99A-*nhaR* in the defined medium without additional NaCl (less than 0.10 M total Na^+^ concentration) and in the presence of 0.75 M NaCl. The wild-type strain MG1655 attained a growth rate of about 0.69 h^-1^ in low NaCl and 0.17 h^-1^ in 0.75 M NaCl. In the absence of IPTG induction, MG1655/pTrc99A-*nhaA* attained similar growth rates and MG1655/pTrc99A-*nhaR* about 20% lower growth rates compared to the wild-type. However, induction of protein expression proved very detrimental to the growth rate of both strains (Figure 1). Even in the absence of additional NaCl, induction of pTrc99A-*nhaA* prevented growth when the IPTG concentration was above 40 μM. Moreover, the growth rates were relatively sensitive to IPTG concentration.

### Overexpression of nhaA and nhaAR using the medium-copy plasmid pBR322

**Figure 2:**
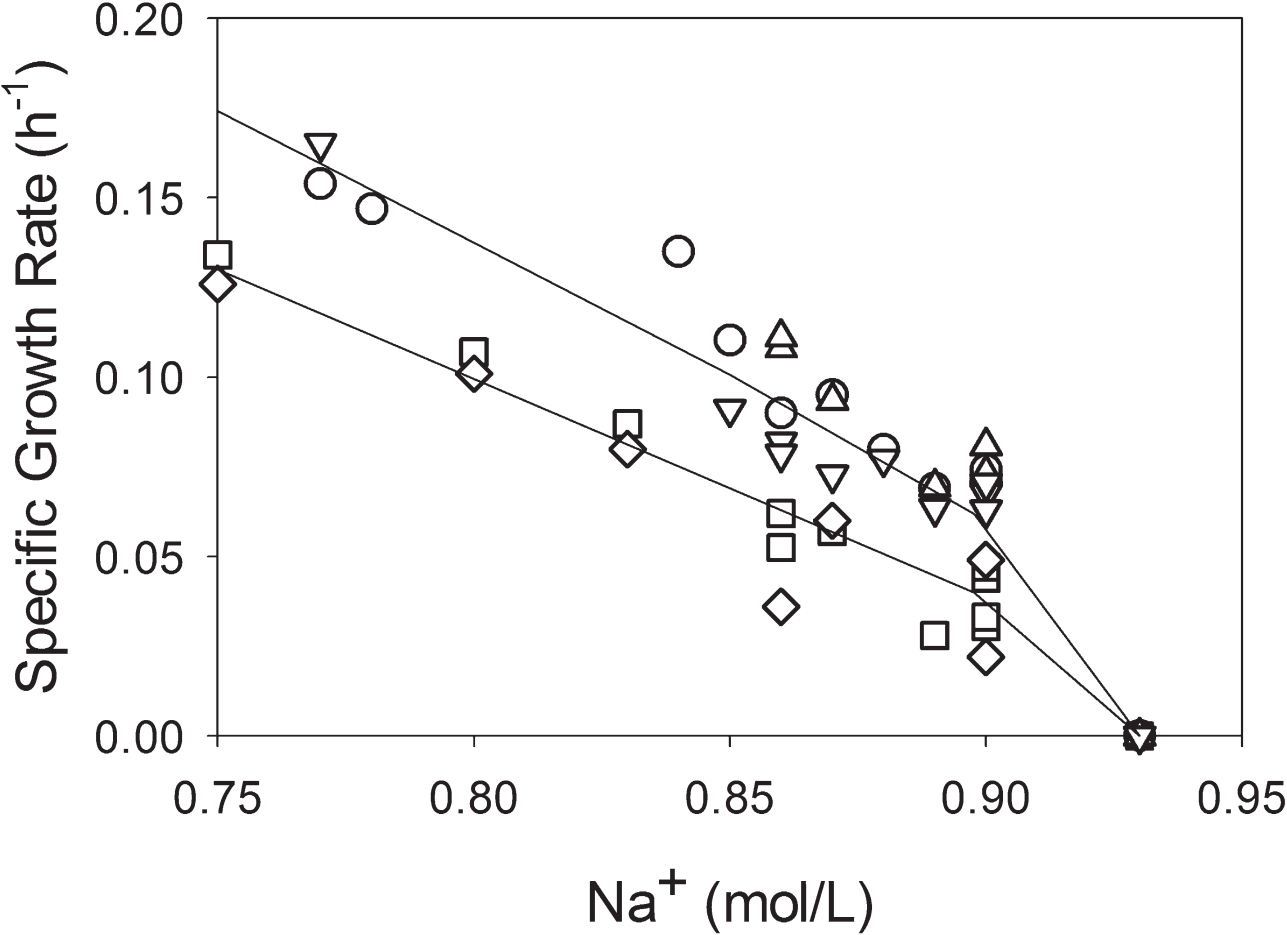
**Maximum specific growth rate (μ**_**MAX**_**) of MG1655 (○), MG1655/pBR322 (□), MG1655/pBR322-*nhaA *(Δ), MG1655/pBR322-*nhaR *(◊), and MG1655/pBR322-*nhaAR *(∇) with increasing total Na**^**+ **^**concentration.**

Because overexpression of *nhaA* or *nhaR* from pTrc99A induced lethality and was very sensitive to expression level—an observation similarly noted by other researchers in the case of *nhaA*[11,12]—we cloned *nhaA*, *nhaR* and *nhaAR* including the relevant promoter(s) into the promoterless pBR322 cloning vector, which exists at about 20 copies per cell [14]. In growth rate experiments with each of the five constructs (MG1655, MG1655/pBR322, MG1655/pBR322-*nhaA*, MG1655/pBR322-*nhaR* and MG1655/pBR322-*nhaAR*) using a defined medium we found that the maximum specific growth rate decreased with increasing Na^+^ concentration (Figure 2). No difference was observed in the maximum specific growth rate for three of the strains over this concentration range (MG1655, MG1655/pBR322-*nhaA*, MG1655/pBR322-*nhaAR*), while MG1655/pBR322 and MG1655/pBR322-*nhaR* showed about 30% lower growth rates. None of the five strains were able to grow in this defined medium containing 0.91 M Na^+^. However, expression of *nhaA* and *nhaR* under these conditions was not detrimental to cell growth in contrast to expression using pTrc99A, even with greatly reduced inducer concentration. Quantitative real time polymerase chain reaction (qPCR) analysis of mRNA levels using both the β-lactamase *bla* gene from pBR322 and the elongation factor Tu *tufA* gene from the chromosome as controls showed that the mRNA levels of *nhaA* increased 8.4-fold in pBR322-*nhaA* and 15.3-fold in pBR322-*nhaAR* with respect to the chromosomal levels, while the mRNA levels of *nhaR* increased 10.5-fold in pBR322-*nhaR* and 29.9-fold in pBR322-*nhaAR* with respect to the chromosomal levels. Interestingly, no increase in *nhaA* mRNA levels was observed in pBR322-*nhaR*.

### Lactate production

Although overexpression of *nhaA*, *nhaR* or *nhaAR* using the pBR322 plasmid did not confer increased Na^+^ tolerance to *E. coli* based on maximum specific growth rate, a beneficial effect of overexpressing these genes might be manifested in other ways. For example, a culture of *E. coli* must be supplied with large quantities of a neutralizing base such as NaOH when the culture accumulates an organic acid. The accumulation of Na^+^ during this process could reduce the rate of organic acid synthesis even though growth might not be affected or might not even be desired to generate the acid product. Since *E. coli* with a knockout of the *pflB* gene encoding pyruvate formate lyase will readily accumulate lactate [10], we next examined the possibility that overexpression of *nhaA, nhaR* or *nhaAR* allowed for greater accumulation of this model acid in *pflB* strains which would not be growing when lactate accumulated. Since pyruvate formate lyase activity is destroyed under aerobic conditions [15], the *pflB* knockout would not be expected to have any impact on aerobic cell growth.

To study the effect of *nhaA*, *nhaR* or *nhaAR* overexpression on lactate accumulation, five constructs were compared including two controls—the *pflB* knockout ALS1317 without the plasmid and ALS1317/pBR322, as well as the *pflB* knockout containing either *nhaA*, *nhaR* or *nhaAR* (ALS1317/pBR322-*nhaA*, ALS1317/pBR322-*nhaR*, or ALS1317/pBR322-*nhaAR*). We first confirmed that overexpression of *nhaA*, *nhaR* or *nhaAR* in ALS1317 had no affect on growth rate compared to MG1655 carrying the identical plasmids (data not shown). Then, these strains were compared with controlled fed-batch bioprocesses in which a low glucose concentration was automatically maintained, and the processes were terminated when glucose was no longer being consumed. The two control strains ALS1317 and ALS1317/pBR322 each achieved 55–56 g/L lactate (Table 1). Similarly, for ALS1317/pBR322-*nhaA* or ALS1317/pBR322-*nhaR* overexpressing *nhaA* or *nhaR* alone, the process was automatically terminated when the concentration reached 58 g/L or less. However, in ALS1317/pBR322-*nhaAR* overexpressing both *nhaA* and *nhaR,* the culture continued to consume glucose until the lactate concentration reached over 70 g/L. Interestingly, the succinate concentration attained was also greater for ALS1317/pBR322-*nhaAR* than for the other constructs: 9.7 g/L versus 3.4–6.3 g/L. The higher total acid concentration was not attained at a greater rate; the lactate productivity for all five different constructs was essentially identical in the range 1.42–1.45 g/L · h. Instead, the presence of both *nhaA* and *nhaR* permitted the prolonged production of lactate. Also, the final Na^+^ concentration for ALS1317/pBR322-*nhaAR* was about 10% greater than for the other constructs.

**Table 1:** **Lactate production and final Na**^**+**^**concentration in fed-batch fermentation**

**Strain**	**Lactate**	**Succinate**	**Final [Na**^**+**^**]**
	**(g/L)**	**(g/L)**	**(mol/L)**
ALS1317	54.5 (1.8)	4.9 (1.3)	0.87 (0.00)
ALS1317/pBR322	56.0 (2.5)	6.0 (0.3)	0.99 (0.02)
ALS1317/pBR322-*nhaA*	57.6 (0.1)	6.3 (0.3)	0.98 (0.01)
ALS1317/pBR322-*nhaR*	55.7 (2.3)	3.4 (0.3)	0.82 (0.01)
ALS1317/pBR322-*nhaAR*	71.2 (0.8)	9.7 (3.7)	1.09 (0.05)

Values indicate mean of replicate fermentations (standard deviations).

## Discussion

In this study using *E. coli*, maximum specific growth rate was initially presumed to serve as an indicator for salt “tolerance”, and growth rate was carefully measured in defined medium over several doubling times. Previous studies have generally used less quantified measures and complex media. Also, previous studies have often involved the *nhaB* gene, encoding for a secondary Na^+^ transporter protein. For example, in order to isolate the gene conferring increased Na^+^/H^+^ antiporter activity, merely growth (+) or no growth (−) was reported for strains in the presence of 0.1 M Li^+^[16]. Padan et al. measured doubling times of *E. coli* in Lysogeny Broth (LB) containing low (0.08 M) and high (0.7 M) Na^+^ of the K12 strain TA15, the Δ*nhaA* derived strain NM81, and NM81 overexpressing *nhaA*[2]. They reported a five-fold increase in doubling time (i.e., a five-fold decrease in growth rate) in the *nhaA* knockout, but insignificant difference between TA15 and NM81-*nhaA*^+^. Pinner et al. studied doubling times of both *nhaA* and *nhaB* knockouts as functions of pH and found *E. coli nhaA nhaB* to be very sensitive to Na^+^, with growth ceasing at 50 mM [7]. The focus of each of these studies was the *decrease* in growth rate as a result of knockouts in *nhaA* and/or *nhaB* and the relative importance of these genes in Na^+^ tolerance, and not whether an elevated growth rate could be obtained by overexpressing either *nhaA* or *nhaB*.

Our studies were based on the simple hypothesis that careful overexpression of *nhaA* and/or *nhaR* should allow *E. coli*, under conditions of greater Na^+^ concentration, to attain a higher growth rate and to accumulate more acid. However, our studies showed elevated expression of *nhaA*, *nhaR* or *nhaAR* did not improve growth rate as a function of Na^+^ concentration. Our results contrast a similar report with *Zymomonas mobilis* showing that elevated *nhaA* expression permitted this strain to grow in complex medium with 0.195 M sodium acetate, whereas no growth was observed using the wild-type control [17]. Expression of the *E. coli nhaA* gene in rice enhanced this plant’s salt tolerance as measured by germination rate, average growth rate of the stem, shoot weight, root weight and rice yield per plant [18]. Also, the *E. coli nhaA* gene expressed in *Saccharomyces cerevisiae* significantly improved yeast growth on agar plates in the presence of Li^+^ but had no effect in the presence of Na^+^[19], while overexpression of the *sod2* gene in *Schizosaccharomyces pombe* increased Na^+^ tolerance as measured by average diameter of colonies on Agar medium [20]. One possible explanation for our results is that while NhaA did enhance Na^+^ extrusion, the growth rate of *E. coli* in defined medium was limited not by extracellular Na^+^ concentration, but by another mechanism such as simple membrane integrity or DNA replication.

Although growth rate was not affected by *nhaA* and/or *nhaR* overexpression, and overexpression of *nhaA* or *nhaR* alone in *E. coli* with a *pflB* knockout did not enhance lactate accumulation compared to controls in a fed-batch fermentation, overexpression of both*nhaA* and *nhaR* permitted 25% greater lactate accumulation. Because we incorporated the complete regulatory region, including both the P1 σ^70^*nhaR*-dependent promoter and the P2 σ^38^*nhaR*-independent promoter of wild-type *nhaA*, which allows *nhaA* to be expressed under a variety of physiological conditions [21], in the pBR322-*nhaA* and pBR322-*nhaAR* plasmids, our observation that pBR322-*nhaAR* allows increased lactate accumulation, whereas pBR322-*nhaA* does not, clearly demonstrates the importance of NhaR in sustaining lactate accumulation by *E. coli* in the presence of increasing Na^+^. Since NhaA is positively regulated by NhaR [22], the specific effect of NhaR could be simply due to the different amounts of NhaA Na^+^/H^+^ antiporter protein produced in these two constructs. Indeed, qPCR results showed greater expression of both *nhaA* and *nhaR* in pBR322-*nhaAR* compared to either pBR322-*nhaA* or pBR322-*nhaR*. Although there is a basal presence of NhaA in wild-type *E. coli*, *nhaA* expression depends on a functional *nhaR* gene [23]. Thus, without the elevated expression of *nhaR*, the level of NhaA produced by pBR322-*nhaA* may be insufficient to allow for increased lactate accumulation. The failure of merely overexpressing *nhaA* or *nhaR* via the high-copy plasmid pTrc99A demonstrates the rigid control necessary for the production of the NhaA membrane protein. Importantly, our results do not preclude an alternative role for NhaR which may be unrelated to NhaA: for example, this gene may impact membrane proteins which are involved in lactate permeation. Nevertheless, elevated levels of both NhaA and NhaR were necessary for enhanced lactate accumulation.

## Conclusions

Overexpression of *nhaA* or *nhaR* had no impact on *E. coli* Na^+^ tolerance as measured by specific growth rate, but the combination of both *nhaA* and *nhaR* did allow *E. coli* to accumulate a 25% higher concentration of lactate. Such a marked increase in final acid titer may benefit the production of other high-volume commercially relevant acids in *E. coli*. The regulation accomplished by *nhaR*, whether to *nhaA* or to some other unknown gene or protein, is therefore critical in allowing cells to Na^+^ tolerance during organic acid generation. Specific growth rate, however, appears be a poor indicator of microbial ‘tolerance’ in the context of its correlation with the accumulation of a biochemical product.

## Methods

### Strains and growth medium

Strains used in this study are listed in Table 2. To construct ALS1317, a P1vir lysate was prepared from NZN111 [24], and the *pflB*:: *Cam* deletion was transduced into MG1655 and chloramphenicol resistant transductant colonies were selected.

**Table 2:** Strains used in this study

**Strains/Plasmids**	**Genotype**	**Notes**
MG1655	*E*. *coli* F- λ- *ilvG rfb*-50 *rph*-1	Wild type
ALS1317	MG1655 *pflB*:: Cam	This study
pTrc99A	Amp^R^*trc*PO *lacI*^*Q*^ ColE1 ori	[13]
pTrc99A-*nhaA*	Amp^R^*trc*PO *lacI*^*Q*^ ColE1 ori *nhaA*	This study
pTrc99A-*nhaR*	Amp^R^*trc*PO *lacI*^*Q*^ ColE1 ori *nhaR*	This study
pBR322	Amp^R^, Tet^R^, ColE1 replicon	[25]
pBR322-*nhaA*	Amp^R^, ColE1 replicon *nhaA*	This study
pBR322-*nhaAR*	Amp^R^, ColE1 replicon *nhaAR*	This study

MG1655 genomic DNA was used as a template with *Pfu* DNA polymerase, and primers were designed based on the published *E. coli* MG1655 genome sequence [26] (GenBank accession number U00096). The *nhaA* coding sequence resides at bases 17,489 through 18,655 on the *E. coli* chromosome while the *nhaR* coding sequence resides just downstream of *nhaA* at bases 18,715 through 19,620. To construct pTrc99A-*nhaA*, the forward primer 5^′^ TACTATGGTACCCAGGAGAACAGCTATGAAACATCTGCATCGATTCTTTAGC 3^′^, which introduced a *Kpn*I restriction site and Shine Dalgarno ribosome binding site just before the start codon of *nhaA*, thus converting the original GTG start codon to an ATG start codon, and the reverse primer 5^′^AGAGAGAGAGAGAGAGAGAGAAGCTTTAACAATGAAAAGGGAGCCGTTTATG 3^′^, which introduced a *Hin*dIII restriction site immediately downstream of the TGA stop codon of *nhaA*, were utilized. The regions of homology to the MG1655 genome are underlined. The amplified 1,265 bp fragment was digested with *Kpn*I and *Hin*dIII and ligated into pTrc99A, which had been digested with the same two restriction enzymes. To construct pTrc99A-*nhaR*, the forward primer 5^′^TACTATGGTACCTTAGGAGGTAACAGCTATGAGCATGTCTCATATCAATTACAAC 3^′^which introduced a *Kpn*I restriction site and Shine Dalgarno ribosome binding site just before the ATG start codon and the reverse primer 5^′^ACACACACACACAAACAACCTGCAGTCGTGGTAATGTACAACTAATCTATCT 3^′^, which introduced a *Pst*I restriction site immediately downstream of the TAA stop codon, were utilized. The regions of homology to the MG1655 genome are underlined. The amplified 1,016 bp fragment was digested with *Kpn*I and *Pst*I and ligated into pTrc99A, which had been digested with the same two restriction enzymes.

As with pTrc99A-*nhaA* and pTrc99A-*nhaR*, to construct pBR322-*nhaA*, pBR322-*nhaR* and pBR322-*nhaAR*, MG1655 genomic DNA was used as a template with *Pfu* DNA polymerase, and primers were designed based on the published *E. coli* MG1655 genome sequence. To construct pBR322-*nhaA*, bases 17,229 through 18,658 of the MG1655 chromosome were amplified using the forward primer 5^′^TACTTTATGAATTCCATCGCCGACTGACAACAAA 3^′^and reverse primer 5^′^TACTTTATCCAAGGCTGTCAAACTGATGGACGCA 3^′^. 261 bases upstream of the start of the *nhaA* coding sequence were included in the amplified region to incorporate both the P1 and P2 promoters for *nhaA*. Dover and Padan showed that *nhaA* is transcribed by both P1, a σ^70^*nhaR*-dependent promoter and P2, a σ^38^*nhaR*-independent promoter and that these promoters are both used under different physiological conditions [21].

An *EcoR*I restriction site was introduced at the beginning of the forward primer, while a *Sty*I restriction site was introduced at the end of the reverse primer. The regions of homology to the MG1655 genome are underlined. To construct pBR322-*nhaR*, bases 17,931 through 19,787 of the MG1655 chromosome were amplified using the forward primer 5^′^TACTTTATGAATTCTTCCGTTAGCGCTGAAGATC 3^′^ and reverse primer 5^′^TACTTTATCCAAGGCTACTCTTTTCAGCATCCCC 3^′^. 784 bases upstream of the start of the *nhaR* coding sequence were included in the amplified region to incorporate the promoter for *nhaR*. This region was shown by Carmel et al. to contain the promoter for *nhaR*[27]. An *EcoR*I restriction site was introduced at the beginning of the forward primer, while a *Sty*I restriction site was introduced at the end of the reverse primer. The regions of homology to the MG1655 genome are underlined.

To construct pBR322-*nhaAR*, bases 17,229 through 19,787 of the MG1655 chromosome were amplified using the forward primer 5^′^TACTTTATGAATTCCATCGCCGACTGACAACAAA 3^′^ and reverse primer 5^′^TACTTTATCCAAGGCTACTCTTTTCAGCATCCCC 3^′^. Just as with pBR322-*nhaA*, 261 bases upstream of the start of the *nhaA* coding sequence were included in the amplified region to incorporate both the P1 and P2 promoters for *nhaA* in pBR322-*nhaAR*. An *EcoR*I restriction site was introduced at the beginning of the forward primer, while a *Sty*I restriction site was introduced at the end of the reverse primer. The regions of homology to the MG1655 genome are underlined. Both PCR products were gel isolated, digested with *EcoR*I and *Sty*I, and ligated into the pBR322 plasmid which had been digested with the same two restriction enzymes.

The defined medium contained (per L): 10 g glucose, 1.70 g citric acid, 13.30 g KH_2_PO_4_, 4.50 g (NH_4_)_2_HPO_4_, 1.2 g MgSO_4_ · 7H_2_O, 13 mg Zn(CH_3_COO)_2_ · 2H_2_O 1.5 mg CuCl_2_ · 2H_2_O, 15 mg MnCl_2_ · 4H_2_O, 2.5 mg CoCl_2_ · 6H_2_O, 3.0 mg H_3_BO_3_, 2.5 mg Na_2_MoO_4_ · 2H_2_O, 100 mg Fe(III) citrate, 4.5 mg thiamine · HCl, and 8.4 mg Na_2_(EDTA) · 2H_2_O. All cultures had an initial pH of 7.0 and were grown in 250 mL baffled shake flasks at 37°C and 250 rpm (19 mm pitch). Na^+^ tolerance was quantified by growing strains in this medium to an optical density (OD) of 2.0, and then transferring the culture into a series of flasks containing the defined medium with additional NaCl to achieve the desired Na^+^ concentration. In these second shake flasks the optical density was measured 5–7 times during exponential growth to calculate the maximum specific growth rate.

### qPCR analysis of mRNA levels

The quantification of *nhaA* and *nhaR* mRNA levels from cells containing the various pBR322 constructs was determined using qPCR with the β-lactamase *bla* gene from pBR322 and the elongation factor Tu *tufA* gene from the chromosome serving as controls. Total RNA was prepared from each pBR322 construct and quantified and then complementary DNA (cDNA) was prepared using RNA primers designed to initiate replication as close to the end of the coding sequence as possible. Amplicons were designed to be approximately 100 bp and forward primers were designed to initiate replication as close to the start codon of the coding sequence as possible. For *nhaA* the 5^′^ACUGAUGGACGCAAACGAAC 3^′^RNA primer was used for cDNA synthesis, while the forward 5^′^TCTTTAGCAGTGATGCCTCG 3^′^and reverse 5^′^TCGTGATACCATCCACTGGT 3^′^DNA primers were used to generate a 100 bp amplicon. For *nhaR* the 5^′^UUAACGCACCGCUGGACUAA 3^′^RNA primer was used for cDNA synthesis, while the forward 5^′^AAAGAAGGTTCCGTGGTTGG 3^′^ and reverse 5^′^TAATTTGCCTTGCAGGCGCT 3^′^DNA primers were used to generate a 102 bp amplicon. For *bla* the 5^′^UGCUUAAUCAGUGAGGCACC 3^′^RNA primer was used for cDNA synthesis, while the forward 5^′^TTCAACATTTCCGTGTCGCC 3^′^ and reverse 5^′^CCCAACTGATCTTCAGCATC 3^′^DNA primers were used to generate a 109 bp amplicon. For *tufA* the 5^′^GAACUUUAGCAACAACGCCC 3^′^RNA primer was used for cDNA synthesis, while the forward 5^′^TGAACGTACAAAACCGCACG 3^′^ and reverse 5^′^GTAGGTTTTAGCCAGTACGG 3^′^DNA primers were used to generate a 103 bp amplicon.

### Fed-batch processes

For lactate production, each of the five constructs (ALS1317, ALS1317/pBR322, ALS1317/pBR322-*nhaA*, ALS1317/pBR322-*nhaR*, or ALS1317/pBR322-*nhaAR*) was first grown in a 250 mL shake flask containing 50 mL of medium. After 12 h the contents were used to inoculate a 2.5 L bioreactor (Bioflo 310, New Brunswick Scientific Co., New Brunswick, NJ, USA) containing 1.0 L of medium with 20 g/L glucose. During an initial aerobic phase of about 6 h the agitation was maintained at 400 rpm, and air and O_2_ were mixed as necessary at a 1.0 L/min total flow rate to maintain the dissolved oxygen above 40% of saturation until the OD reached 8.0. During a second, anaerobic phase the agitation was maintained at 200 rpm, and a 9:1 mixture of N_2_ and CO_2_ was sparged at 0.5 L/min. During this phase the glucose concentration was maintained at 2–4 g/L using a 600 g/L glucose solution automatically fed in response to the measurement of an on-line glucose analyzer (YSI 2700 SELECT, YSI Life Sciences Inc., Yellow Springs, OH, USA). Each fed-batch experiment was terminated when the glucose solution was not demanded for 3 h. For both phases, the pH was controlled at 7.0 using 30% (w/v) NaOH, and the temperature at 37°C. All fermentations were run in duplicate.

### Analytical methods

The optical density at 600 nm (OD) (UV–650 spectrophotometer, Beckman Instruments, San Jose, CA, USA) was used to monitor cell growth. Concentrations of soluble organic compounds were determined by high performance liquid chromatography as previously described [28].

## Competing interests

The authors declare that they have no competing interests.

## Authors’ contributions

ME and EA conceived the study. XW, RA, ME and EA designed components of the study and interpreted the results. RA completed the molecular biology aspects of this work. XW carried out the fermentations and acquired the data. XW prepared the manuscript draft. All authors read and approved the final manuscript.
